# Effects of Nitrogen and Phosphorus Addition on Soil Extracellular Enzyme Activity and Stoichiometry in Chinese Fir (*Cunninghamia lanceolata*) Forests

**DOI:** 10.3389/fpls.2022.834184

**Published:** 2022-03-09

**Authors:** Meihua Liu, Bingping Gan, Quan Li, Wenfa Xiao, Xinzhang Song

**Affiliations:** ^1^State Key Laboratory of Subtropical Silviculture, Zhejiang A&F University, Hangzhou, China; ^2^Research Institute of Forest Ecology, Environment and Protection, Chinese Academy of Forestry, Beijing, China

**Keywords:** nitrogen deposition, phosphorus, soil enzymes, enzyme stoichiometry, Chinese fir

## Abstract

Soil extracellular enzymes play an important role in microbial functions and soil nutrient cycling in the context of increasing N deposition globally. This is particularly important for Chinese fir (*Cunninghamia lanceolata*) forests because of the decline in soil fertility induced by successive rotation. In this study, we aimed to determine the effects of simulated N deposition (N30: 30 kg ha^−2^ year^−1^; N60: 60 kg ha^−2^ year^−1^) and phosphorus addition (P20: 20 mg kg^−1^; P40: 40 mg kg^−1^) on the activity and stoichiometry of soil extracellular enzymes related to soil C, N, and P cycling in Chinese fir. The results showed that N addition alone increased the activity of soil β-1,4 glucosidase (BG) but decreased the activity of *N*-acetyl-β-d-glucosidase (NAG) and leucine aminopeptidase (LAP). N addition increased the ratios of soil enzymes, C:N and C:P, alleviated microbial N-limitation, and aggravated microbial C-limitation. P addition alone increased enzyme activity, and P40 addition increased the ratio of BG to soil microbial biomass carbon (MBC), and (NAG + LAP):MBC activity ratio, thereby aggravating C restriction. N and P co-addition significantly affected soil extracellular enzyme activity and stoichiometry. For instance, BG activity and BG:MBC activity ratio increased significantly under the N30 + P40 treatment, which intensified C-limitation. Soil pH was the main factor influencing enzyme activity, and these variables were positively correlated. The stoichiometric relationships of enzyme reactions were coupled with soil pH, total nitrogen (TN), and available phosphorus (AP). Our results indicate that changes in soil characteristics induced by N and P inputs influence the activities of soil microorganisms and result in changes in microbial resource acquisition strategies. This study provides useful insights into the development of management strategies to improve the productivity of Chinese fir forests under scenarios of increasing N deposition.

## Introduction

In recent decades, nitrogen (N) deposition in the atmosphere has increased dramatically owing to the burning of fossil fuels and the extensive use of N-based fertilizers. Global atmospheric N deposition has been predicted to increase to 200 Tg N year^−1^ by 2050 (Galloway et al., 2008; Moorhead et al., 2013). Excessive N deposition negatively affects the N cycle and balance (Vitousek et al., 2010), influencing soil nutrient status, soil microbial diversity, and vegetation productivity (Ullah et al., 2019; Song et al., 2020). In addition, phosphorus (P) is one of the most important elements in nature and is involved in the synthesis of biological cell membranes and the activation of enzymes (Vance et al., 2003). Soil P is easily chemically bound to aluminum (Al) and iron (Fe) oxides and hydroxides present in soil, which strongly inhibits its release from soils, resulting in low P utilization (Lin et al., 2020). Dramatically increased N deposition may aggravate (Vitousek et al., 2010; Dong et al., 2019) or alleviate (Li et al., 2021a) soil P limitation, which can further influence plant growth and ecosystem carbon (C) uptake (Liang et al., 2020; Song et al., 2020).

Soil extracellular enzymes play a key role in microbial functions and soil nutrient cycling (Sinsabaugh et al., 2005; Xu et al., 2017) and are important indicators of microbial nutrient demand and organic matter decomposition (Caldwell, 2005). These enzymes are directly involved in the transformation of soil substances as well as nutrient release (Kelley et al., 2011; Burns et al., 2013). For example, soil extracellular enzymes are involved in the degradation of nucleic acids, proteins, and phospholipids (Sinsabaugh et al., 2008). The soil enzymes β-1,4 glucosidase (BG), β-d-cellobiohydrolase (CBH), and β-1,4 xylosidase (βX) are involved in the C cycle, and leucine aminopeptidase (LAP) and *N*-acetyl-β-d glucosamine (NAG) contribute to the N cycle, whereas alkaline phosphatase (AKP) and acid phosphatase (ACP) are responsible for the P cycle (Caldwell, 2005; Sinsabaugh et al., 2008). Notably, numerous studies have shown that soil nutrient availability is significantly correlated with enzyme activity (Zheng et al., 2015; Guan et al., 2020; Bai et al., 2021). For example, N fertilizers can promote soil respiration and hydrolytic enzyme activity but inhibit soil oxidase activity (Li et al., 2018) and reduce the activity of phenoloxidase and peroxidase (Jian et al., 2016). Increasing P fertilizer application can significantly reduce soil phosphatase activity (Zheng et al., 2015) and affects β-glucosidase, N-acetyl-glucosaminidase, and acid and alkaline phosphomonoesterase activity (Wang et al., 2020). Therefore, soil extracellular enzyme activity (EEA) depends on the physicochemical properties of the soil (Burns et al., 2013). A more detailed characterization of soil enzymes and their responses to N deposition is needed to understand the mechanisms of forest-soil feedbacks to increase N deposition globally.

Many studies of EEA (Aragón et al., 2014; Hill et al., 2018; Hu et al., 2021) have examined the ecological stoichiometric relationships between soil enzymes (Sinsabaugh et al., 2008; Xu et al., 2021). Notably, EEA is related to microbial nutrient utilization and the availability of soil nutrients (Hill et al., 2012), which can be used to explore key ecological processes for nutrient dynamics. The activities of different enzymes, such as BG, NAG, LAP, and ACP, are often used as indicators of C, N, and P acquisition (Sinsabaugh et al., 2008; Peng and Wang, 2016; Chen et al., 2018a). Furthermore, extracellular enzyme stoichiometry (EES) reflects the equilibrium between the microbial biomass and organic matter elemental composition of the soil (Sinsabaugh et al., 2009; Sinsabaugh and Follstad, 2012) and can indicate the nutrient utilization strategy of microorganisms (Hill et al., 2012). For example, the BG:ACP activity ratio is related to soil P content and is negatively correlated with P availability (Allison and Vitousek, 2005), while the ratio of BG:(NAG + LAP) activity increases with soil available N (Guan et al., 2020). Therefore, soil enzymes can mediate nutrient cycling between soils and plants and reflect environmental nutrient availability. Furthermore, studies of soil EES can contribute to a better understanding of how N deposition and P addition influence soil microbial demand for C, N, and P resources, providing insights into the influence of microbial mechanisms on nutrient cycling (Zhou et al., 2017).

Chinese fir (*Cunninghamia lanceolata*) is a subtropical, fast-growing timber species with the largest plantation area of artificial forests in China. However, due to successive rotation, these forests face a number of management problems including declines in productivity, soil fertility (Miao et al., 2019), and biodiversity (Xu et al., 2020). Previous studies have shown that these declines might be related to the low soil nutrient availability (Ma et al., 2007; Wu et al., 2012). Most of the soils in the distribution area of the Chinese fir forest are P-deficient due to successive rotations (20–25 years for each Chinese fir rotation period; Wu et al., 2011). Concurrently, the average N deposition in subtropical China has increased in recent decades. Increasing soil P deficiency and N excess have become the dominating factors that limit the high productivity in Chinese fir. As soil enzymes play a vital role in the decomposition of soil substances and nutrient cycling, the mechanism by which soil enzyme activity responds to soil nutrient limitation under scenarios of N deposition needs to be further explored. In this study, we focused on the stoichiometry of soil enzyme activity in Chinese fir forests under simulated N deposition and P addition. In addition to detecting patterns in soil enzyme activity, stoichiometry, and soil physicochemical properties, we evaluated (1) the effects of N and P additions on the EEA ratios of various soil enzymes involved in C, N, and P cycling, and (2) the effects of N and P additions on the relative nutrient limitation of the soil. We sought to provide useful insights into the potential improvement of plantation productivity and management of Chinese fir forests under scenarios of increasing N deposition.

## Materials and Methods

### Field Experiment

The experimental sites were established in Gaokan Village, Lin’an District, Hangzhou City (119°67′E, 30°21′N), Zhejiang Province, China, with an annual average precipitation of 1,420 mm and annual average temperature of 15.6°C (Li et al., 2019). The local N deposition rate is 30.9 kg ha^−2^ year^−1^ (Song et al., 2015). A Chinese fir plantation was established here in 2007. The field experiments were conducted in January 2017, as the average tree height was approximately 3 m and the average diameter at breast height (measured at 1.3 m above ground level) was 12–14 cm. Twenty-seven plots (3 × 3 m each) were selected with a distance of at least 6 m between each plot, and there was one *C. lanceolata* tree at the center of each plot. Each treatment was performed in three individual plots as three independent replicates as previously described (Liu et al., 2019, 2021). Briefly, the plots were subject to a control treatment (no added N, N0, and no added P, P0, or N0 + P0) or eight different nutrient levels, as follows: no added N (N0), low N addition of 30 kg N ha^−1^ year^−1^ (N30), and high N addition of 60 kg N ha^−1^ year^−1^ (N60); no P addition (P0), low P addition of 20 mg P kg^−1^ (P20), and high P addition of 40 mg P kg^−1^ (P40); N30 with low or high P treatment (N30 + P20 and N30 + P40); and N60 treated with low or high P (N60 + P20 and N60 + P40).

In January 2017, after 30-cm deep plowing in each plot, calcium and magnesium phosphate fertilizers were sprayed uniformly to ensure that the soil available phosphorus (AP) concentration in the upper layer of the soil reached the required level (20 or 40 mg P kg^−1^). Between April 2017 and April 2020, to achieve the required N deposition level (30 or 60 kg N ha^−1^ year^−1^), N fertilizers were evenly sprayed with dissolved NH_4_NO_3_ from the top of the trees in each plot at the beginning of each month, for a total of 36 equal applications. The trees in the control treatment were sprayed with the same volume of nitrogen-free water. All the plots were subject to the same environmental factors.

### Field Sampling

Soil samples were collected in April 2020. In each plot, nine soil cores measuring 3 cm in diameter and at a depth of 25 cm were collected randomly from around the roots of a selected tree and were manually homogenized as the soil sample of the selected tree. Fresh soil samples were transported to the laboratory and sieved through a 2-mm mesh to remove coarse material before further analysis. Some of the soil samples were frozen at −20°C to analyze soil water content (SWC) and soil microbial biomass carbon (MBC). The other soil samples were air-dried at room temperature (22–28°C) for a week for the analysis of BG, NAG, LAP, and ACP enzyme activity and soil physicochemical properties, such as soil pH, soil available nitrogen (AN), soil AP, soil total nitrogen (TN), and total phosphorus (TP) content.

### Soil Physiochemical Properties

Soil moisture content (SMC) was determined as the mass loss after drying the isolates at 105°C for 24 h (de Knegt and van den Brink, 1998). Soil pH was determined in a 1:2.5 (*w*/*v*) soil-to-water extract using a digital pH meter (FE20, Mettler Toledo, Switzerland). Soil AP was extracted using the diacid method and quantified using the molybdenum-antimony colorimetric method (Murphy and Riley, 1962). The concentration of AN was analyzed using the NaOH hydrolysis diffusion method (Xiao et al., 2018). AP was analyzed using the molybdenum blue method (Watanabe and Olsen, 1965). Soil organic carbon (SOC), TP, and TN were determined using an elemental analyzer (ElementarVario EL III, Germany).

### Microbial Biomass Carbon and Soil Enzyme Activity Analysis

Microbial biomass carbon was determined using the chloroform (CHCl_3_) fumigation extraction method (Brookes et al., 1985; Vance et al., 1987). Soluble organic C was extracted with 0.5 M K_2_SO_4_ with a soil:solution ratio of 1:3 (*w*/*v*) before and after 24 h of CHCl_3_ fumigation. After filtration with medium-speed quantitative filter paper, the extractable organic C in the soil extracts was analyzed using a TOC analyzer (Elementar Vario EL III, Germany). MBC was calculated as the difference in organic C concentrations between non-fumigated and fumigated soils, and an efficiency factor of 0.45 was used to correct for incomplete extraction.

Soil extracellular enzyme activity (BG, NAG, LAP, and ACP) was assayed using a colorimetric 96-well microtiter plate assay based on the increase or decrease in the substrate concentration (Tabatabai and Bremner, 1969; Cui et al., 2015). Briefly, a soil suspension was prepared by homogenizing 0.02 g of naturally air-dried soil with 10 μl of toluene. Subsequently, 130 μl of 0.05 mM p-nitrophenyl-β-d-glucopyranoside (for measuring BG) or 4-nitrophenol-β-N-acetylglucosamine (for measuring NAG) was added to the soil suspension, and the same amount of distilled water was added to the control group. Thereafter, citrate phosphate buffer (pH = 6.0) was added and mixed with the solution. After soaking at 37°C for 1 h and water bathing at 90°C for 5 min, the solution was centrifuged for 10 min at 25°C. Subsequently, 130 μl of 0.1 mM Na_2_CO_3_ solution was added to the supernatant for 2 min. Fluorescence was measured using a microplate fluorometer (Spectramax 190, United States) at 400 nm. For LAP measurements, 0.05 g of air-dried soil was weighed into 2-ml centrifuge tubes and mixed with a pH 7.2 modified universal buffer (Tris-HCl). The indicator substrate, 0.05 mM leucyl p-nitroanilide, was then added to the reaction system, followed by 1 h incubation at 37°C. The homogenate was centrifuged at 8,000 × *g* for 10 min at 4°C, and the absorbance of the supernatant was measured at 405 nm.

ACP activity was determined by measuring the release of p-nitrophenol from p-nitrophenyl phosphate (PNPP) as described by Tabatabai and Bremner (1969). Briefly, 0.1 g of air-dried soil was weighed into 50 μl toluene and 0.4 ml of 0.05 mM PNPP followed by 24 h incubation at 37°C. Then, a 1 ml mixture of 0.01 mM CaCl_2_ and 0.04 mM NaOH was added to stop the reaction. The homogenate was centrifuged at 8,000 *g* for 10 min at 25°C, and the PNP in the supernatant was measured using spectrophotometry at 405 nm.

### Soil Microbial Element Limitation

We tested common stoichiometric indicators to estimate the soil microbial element limitation. The nutrient distribution status of each enzyme synthesized by microorganisms was expressed as the ratio of enzyme activity to MBC. According to Allison et al. (2010), this ratio reflects the status of soil nutrients and changes in the nutrient acquisition strategy of microorganisms. Sinsabaugh et al. (2009) showed that the activity of C, N, and P acquisition enzymes in all habitats is close to 1:1:1. When the nutrients that microorganisms can directly use in the soil are limited, corresponding enzymes are secreted to obtain nutrients (Allison and Vitousek, 2005), which changes the stoichiometry of the soil enzymes. The enzyme C:N, C:P, and N:P ratios were calculated as follows (Dong et al., 2019):


$$E_{C:N}=\ln\left(\mathrm{BG}\right):\ln\left(\mathrm{NAG}+\mathrm{LAP}\right)$$

$$E_{C:P}=\ln\left(\mathrm{BG}\right):\ln\left(\mathrm{ACP}\right)$$

$$E_{N:P}=\ln\left(\mathrm{NAG}+\mathrm{LAP}\right):\ln\left(\mathrm{ACP}\right)$$

Vector analysis of enzyme activity (vector length and vector angle) was used to test the relative nutrient limits, where a relatively long vector length indicates a larger C limit, and a vector angle of <45° or >45° indicates the relative nutrient limits of N or P, respectively. The calculation formulas of vector length (VL, dimensionless) and vector angle (VA, degrees) are as follows (Dong et al., 2019):


$$\mathrm{VL}={\left[{\left(\ln\left(\mathrm{BG}\right):\ln\left(\mathrm{NAG}+\mathrm{LAP}\right)\right)}^{2}+{\left(\ln\left(\mathrm{BG}\right):\ln\left(\mathrm{ACP}\right)\right)}^{2}\right]}^{1/2}$$

$$\mathrm{VA}=Degrees\left(ATAN2\left(\begin{array}{l} \left(\ln\left(\mathrm{BG}\right):\ln\left(\mathrm{ACP}\right)\right),\\ \left(\ln\left(\mathrm{BG}\right):\ln\left(\mathrm{NAG}+\mathrm{LAP}\right)\right) \end{array}\right)\right)$$

### Statistical Analysis

Data analysis was performed using SPSS (version 20.0; SPSS Inc. Chicago, IL, United States). One-way analysis of variance (ANOVA) and least significant difference (LSD) tests were used to compare pH, SWC, SOC, TN, TP, AN, AP, BG, NAG + LAP, ACP enzyme activity, and stoichiometry under the different treatments. A two-factor ANOVA was used to evaluate the combined influence N and P addition on the experimental variables. The Pearson correlation coefficient was used to analyze the correlation between soil physical and chemical properties, enzyme activity, and enzyme stoichiometry. Redundancy analysis (RDA) was carried out using Canoco5 software (Braak and Smilauer, 2012) to evaluate the impact of environmental factors (soil physical and chemical properties) on the activity of the various soil enzymes, VA, VL, and enzyme extracellular activity ratios. All figures were drawn using Origin Pro 2018 (Origin Lab Corporation).

## Results

### Changes in Soil Physicochemical Properties

The interaction of N addition, P addition, and N + P addition significantly affected soil pH, SOC, AN, and AP (Table 1). Compared with the control, N application significantly reduced soil pH (Table 2). N30 addition significantly reduced soil SOC content by 37.79% (*p* < 0.05) and N60 addition significantly increased AN and AP content by 14.99 and 28.09% (*p* < 0.05), respectively. P20 addition significantly reduced soil SOC content by 23.05% (*p* < 0.05), and P40 addition significantly reduced TN content but significantly increased soil pH, TP, and AP content. Under the N30 treatment, P addition significantly increased pH, SOC, and AN content. In comparison, under the N60 treatment, P addition significantly increased AP content (*p* < 0.05). Under the P20 treatment, N addition significantly increased soil SOC and AN content, while under the condition of P40 treatment, N application significantly reduced pH, AP, and TP content and significantly increased AN content (*p* < 0.05).

**Table 1:** Two-way ANOVA of the effects of simulated nitrogen (N) deposition and phosphorus (P) addition on soil nutrient concentration, soil enzyme activity, and soil enzyme stoichiometry of Chinese fir.

Variables/factors	N addition		P addition		N + P addition	
	*F*-value	*p* value	*F*-value	*p* value	*F*-value	*p* value
Soil pH	486.96	0.000^***^	10.88	0.001^**^	11.69	0.000^***^
Soil SWC	3.72	0.044^*^	1.54	0.242	1.28	0.314
Soil SOC	5.36	0.015^*^	11.24	0.001^**^	15.27	0.000^***^
Soil TN	25.71	0.000^***^	0.36	0.702	6.73	0.002^**^
Soil AN	80.34	0.000^***^	39.45	0.000^***^	18.91	0.000^***^
Soil TP	4.93	0.020^*^	57.57	0.000^***^	2.60	0.071
Soil AP	7.69	0.004^**^	285.39	0.000^***^	37.61	0.000^***^
BG	76.30	0.000^***^	16.75	0.000^***^	24.51	0.000^***^
NAG + LAP	54.86	0.000^***^	15.76	0.000^***^	9.06	0.000^***^
ACP	9.88	0.001^**^	15.23	0.000^***^	2.93	0.050
E_C:N_	5.25	0.016^*^	3.65	0.047^*^	18.08	0.000^***^
E_C:P_	7.58	0.004^**^	16.38	0.000^***^	0.70	0.603
E_N:P_	2.54	0.107	10.06	0.001^**^	4.54	0.010^*^
VL	10.04	0.001^**^	19.17	0.000^***^	1.95	0.146
VA	2.65	0.098	10.15	0.001^**^	4.36	0.012^*^

SWC, soil water content; SOC, soil organic carbon; TN, total nitrogen; AN, available nitrogen; TP, total phosphorus; AP, available phosphorus; BG, β-glucosidase; NAG + LAP, the sum of soil *N*-acetyl-β-d-glucosidase and leucine aminopeptidase; ACP, acidic phosphomonoesterase; E_C:N_, ln(BG):ln(NAG + LAP); E_C:P_, ln(BG):ln(ACP); and E_N:P_, ln(NAG + LAP):ln(ACP). Repeatable two-way ANOVA was used to statistically test.

* *p* < 0.05;

** *p* < 0.01;

*** *p* < 0.001.

**Table 2:** Soil physicochemical properties under simulated nitrogen (N) deposition and phosphorus (P) addition.

Treatments	pH	SWC (%)	SOC (g kg^−1^)	TN (g kg^−1^)	AN (mg kg^−1^)	TP (g kg^−1^)	AP (mg kg^−1^)	Soil C:N	Soil C:P	Soil N:P
N0 + P0	4.99 ± 0.02b	27.64 ± 1.30b	41.65 ± 2.94b	1.48 ± 0.07bc	88.05 ± 1.29d	0.17 ± 0.01c	13.60 ± 0.99g	28.02 ± 0.76ab	245.24 ± 11.24bc	8.75 ± 0.27b
P20	5.02 ± 0.03b	26.21 ± 1.73b	32.05 ± 1.01c	1.28 ± 0.10c	88.31 ± 1.07d	0.15 ± 0.01c	15.51 ± 0.64f	25.29 ± 1.22b	210.70 ± 10.60c	8.33 ± 0.14b
P40	5.16 ± 0.02a	28.29 ± 2.76ab	37.59 ± 3.60bc	1.17 ± 0.12c	82.71 ± 5.36d	0.55 ± 0.08a	30.83 ± 0.28a	32.33 ± 2.79a	69.66 ± 4.18e	2.17 ± 0.16d
N30	4.79 ± 0.02d	28.24 ± 1.36ab	25.91 ± 4.29c	1.52 ± 0.01b	95.16 ± 1.54c	0.11 ± 0.01c	14.88 ± 0.58fg	17.11 ± 2.91c	227.27 ± 21.48c	13.74 ± 1.51a
N30 + P20	5.07 ± 0.02b	30.85 ± 0.97ab	50.21 ± 1.64a	1.81 ± 0.08a	146.38 ± 1.45a	0.15 ± 0.01c	14.92 ± 0.43fg	27.72 ± 0.31b	343.52 ± 9.80a	12.39 ± 0.23a
N30 + P40	4.90 ± 0.02c	32.44 ± 1.13a	50.48 ± 0.63a	1.92 ± 0.10a	112.58 ± 3.02b	0.33 ± 0.02b	25.03 ± 0.77b	26.36 ± 1.05b	152.70 ± 8.70d	5.79 ± 0.11c
N60	4.37 ± 0.02e	30.95 ± 1.02ab	38.84 ± 1.89bc	1.58 ± 0.05b	101.25 ± 2.48c	0.16 ± 0.02c	17.42 ± 0.61e	24.62 ± 0.55b	245.36 ± 14.35bc	9.98 ± 0.67b
N60 + P20	4.39 ± 0.05e	27.67 ± 0.75b	46.18 ± 1.07ab	1.57 ± 0.04b	112.98 ± 5.30b	0.17 ± 0.02c	19.88 ± 0.36d	29.42 ± 0.47ab	269.19 ± 16.81b	9.15 ± 0.56b
N60 + P40	4.31 ± 0.03e	30.09 ± 0.96ab	43.78 ± 1.42ab	1.41 ± 0.05bc	101.51 ± 2.44c	0.40 ± 0.07b	22.38 ± 0.40c	31.05 ± 0.11ab	114.96 ± 13.66d	3.70 ± 0.43d

N0, 0 kg N ha^−2^ year^−1^; N30, 30 kg N ha^−2^ year^−1^; N60, 60 kg N ha^−2^ year^−1^; P0, 0 mg P kg^−1^; P20, 20 mg P kg^−1^; P40, 40 mg P kg^−1^; SWC, soil water content; SOC, soil organic carbon; TN, total nitrogen; AN, available nitrogen; TP, total phosphorus; AP, available phosphorus; Soil C:N, the ratio of soil SOC to TN; Soil C:P, the soil of SOC to TP; and Soil N:P, the soil of TN to TP. The value is the mean ± standard error (*n* = 27). Different letters in the same column indicate that there are significant differences between different treatments based on one-way ANOVA followed by LSD test (*p* < 0.05).

### Changes in Soil MBC and EEA

**Figure 1:**
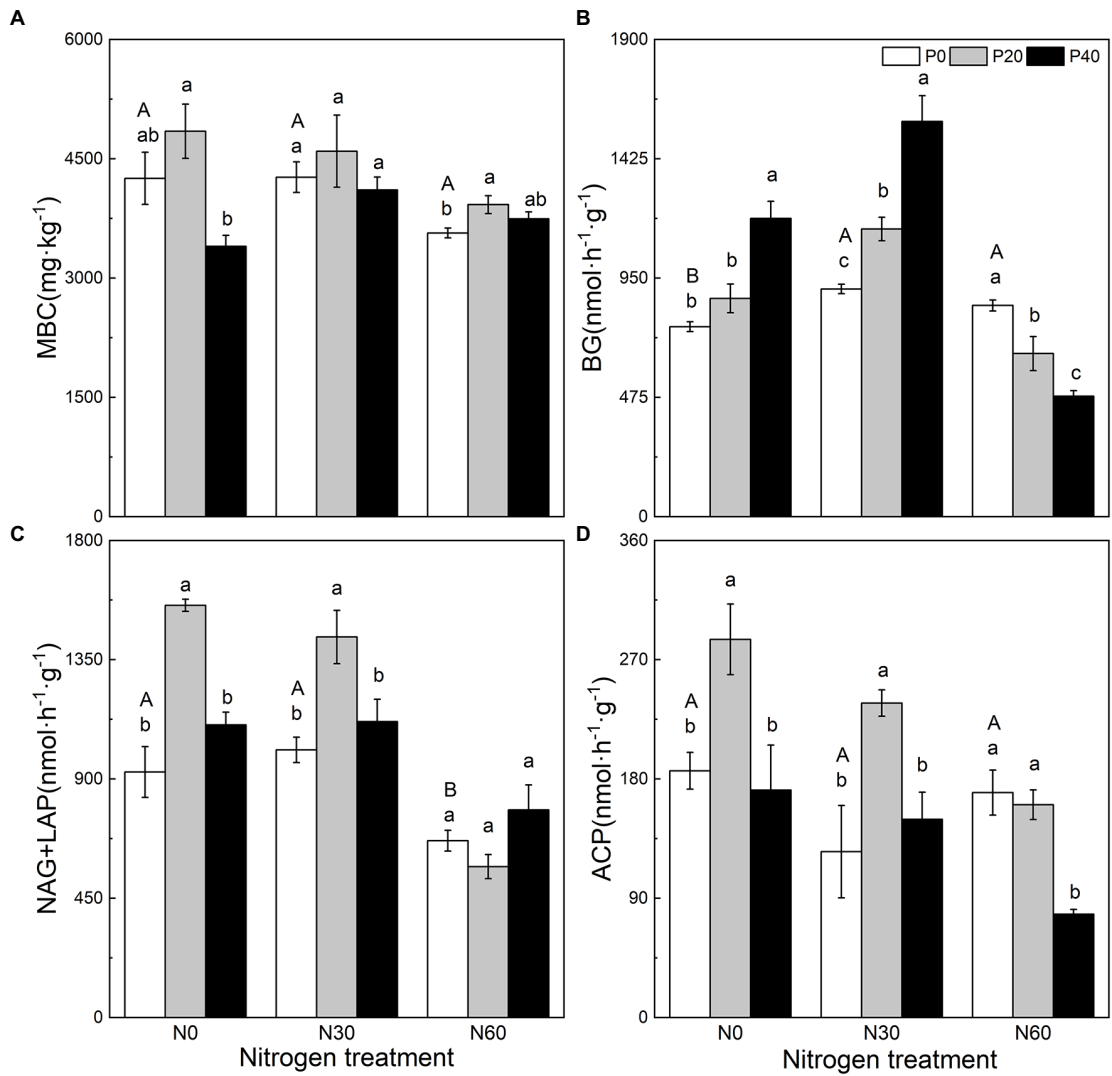
The effect of simulated nitrogen (N) deposition and phosphorus (P) addition on microbial carbon content and soil enzyme activity. **(A)** Soil microbial biomass carbon. **(B)** The activity of soil β-glucosidase. **(C)** The activity of soil N-acetyl-β-D-glucosidase and soil leucine aminopeptidase. **(D)** The activity of soil acid phosphatase. N0, 0 kg N ha^−2^ year^−1^; N30, 30 kg N ha^−2^ year^−1^; N60, 60 kg N ha^−2^ year^−1^; P0, 0 mg P kg^−1^; P20, 20 mg P kg^−1^; P40, 40 mg P kg^−1^; MBC, soil microbial biomass carbon; BG, Soil β-glucosidase; NAG + LAP, the sum of soil *N*-acetyl-β-d-glucosidase and soil leucine aminopeptidase; and ACP, soil acid phosphatase. Different lowercase letters indicate significant differences between P addition rates at identical N addition rates. Different capital letters indicate significant differences between N addition rates at identical P addition rates (*p* < 0.05).

N addition, P addition, and the interaction of N + P addition affected soil MBC, BG activity, NAG + LAP activity, and ACP activity (Figure 1). Compared with the control, the P20 treatment significantly increased NAG + LAP and ACP activities (*p* < 0.05), while P40 treatment significantly increased BG activity (*p* < 0.05; Figure 1). Under the N30 treatment, P20 addition significantly increased BG, NAG + LAP, and ACP activities (*p* < 0.05), and P40 application significantly increased BG activity (*p* < 0.05). Under the N60 treatment (*p* < 0.05), P20 significantly increased MBC, P application significantly reduced BG activity (*p* < 0.05), and the P40 treatment significantly reduced ACP activity (*p* < 0.05).

**Figure 2:**
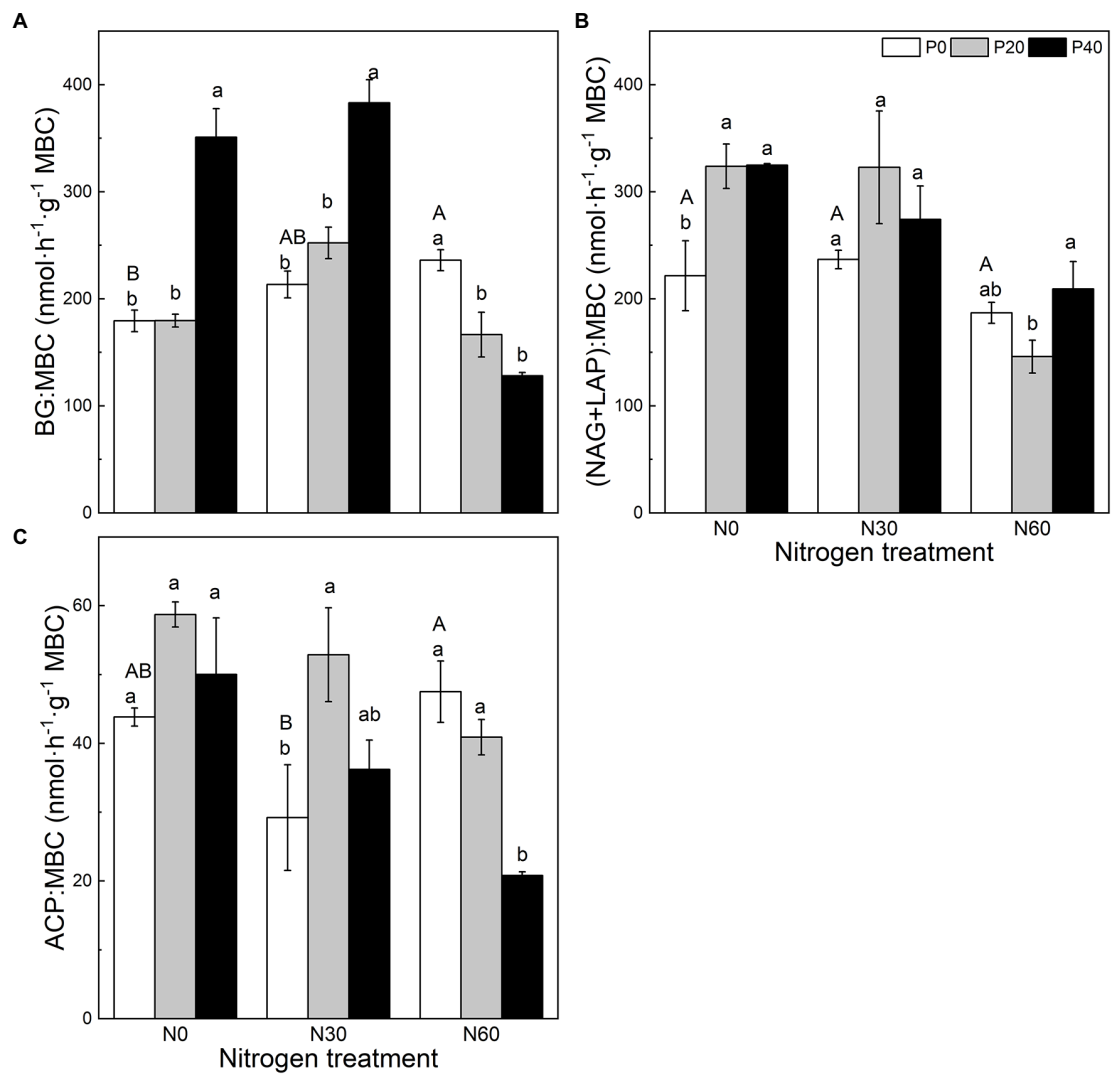
The effect of simulated nitrogen (N) deposition and phosphorus (P) addition on the nutrient distribution of enzymes. **(A)** The ratio of BG activity to MBC. **(B)** The ratio of NAG and LAP activity to MBC. **(C)** The ratio of ACP activity to MBC. N0, 0 kg N ha^−2^ year^−1^; N30, 30 kg N ha^−2^ year^−1^; N60, 60 kg N ha^−2^ year^−1^; P0, 0 mg P kg^−1^; P20, 20 mg P kg^−1^; P40, 40 mg P kg^−1^; BG, Soil β-glucosidase; NAG + LAP, the sum of soil *N*-acetyl-β-d-glucosidase and soil leucine aminopeptidase; ACP, soil acid phosphatase; and MBC, soil microbial biomass carbon. Different lowercase letters indicate significant differences between P addition rates at identical N addition rates. Different capital letters indicate significant differences between N addition rates at identical P addition rates (*p* < 0.05).

Compared with the control, under the P40 treatment, the BG:MBC ratio increased significantly (*p* < 0.05; Figure 2). A combined treatment of N30 and P20 significantly increased the ACP:MBC ratio (*p* < 0.05), and the combined addition of N30 and P40 significantly increased the BG:MBC ratio (*p* < 0.05). Under the N60 treatment, P application significantly reduced the BG:MBC ratio (*p* < 0.05), while P40 addition significantly reduced the ACP:MBC ratio (*p* < 0.05).

### Soil Enzyme Stoichiometric Ratio and Relative Nutrient Limitation

**Figure 3:**
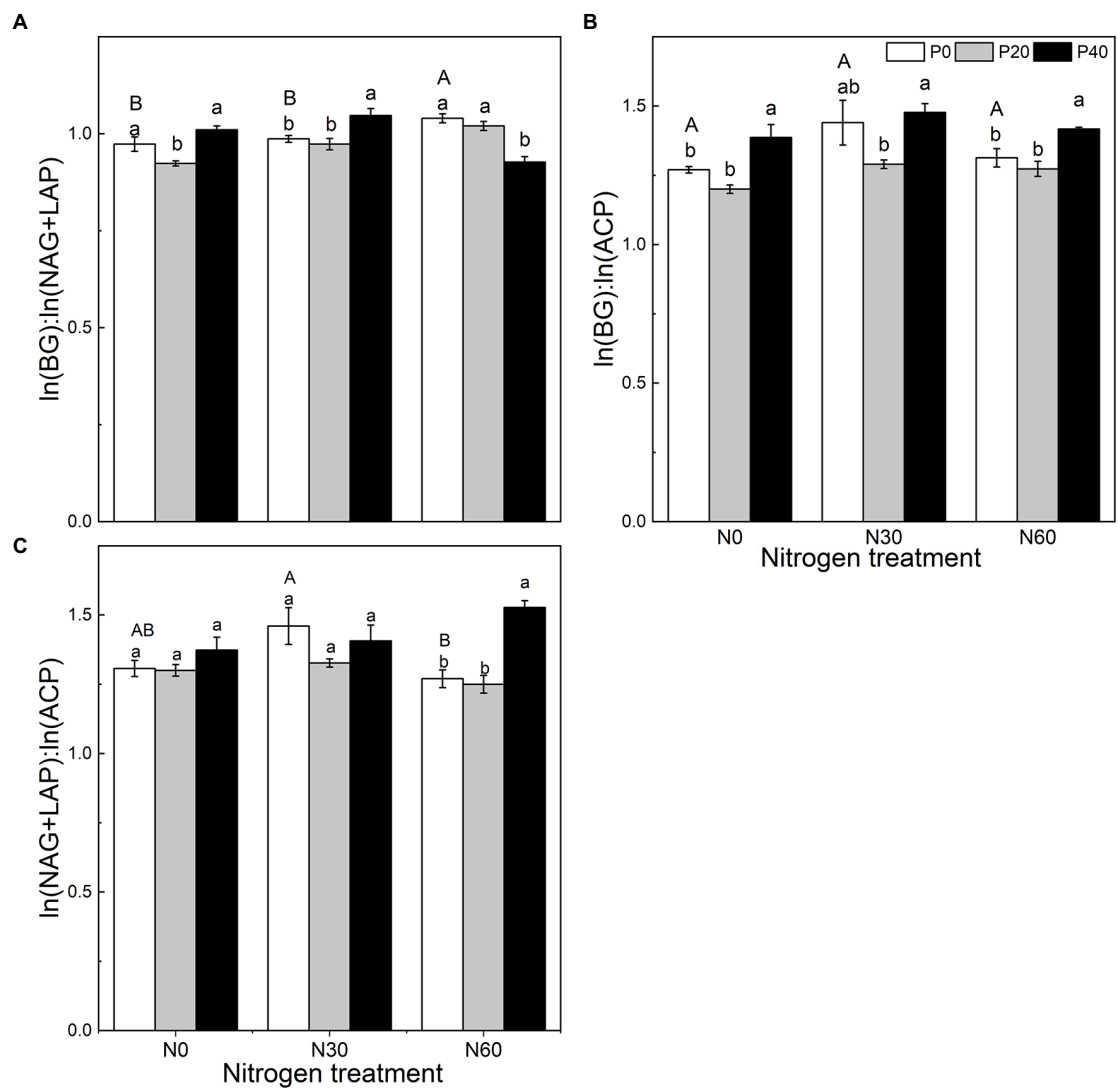
The effect of simulated nitrogen (N) deposition and phosphorus (P) addition on the stoichiometric ratio of soil enzymes. **(A)** The enzyme C:N ratio. **(B)** The enzyme C:P ratio. **(C)** The enzyme N:P ratio. N0, 0 kg N ha^−2^ year^−1^; N30, 30 kg N ha^−2^ year^−1^; N60, 60 kg N ha^−2^ year^−1^; P0, 0 mg P kg^−1^; P20, 20 mg P kg^−1^; P40, 40 mg P kg^−1^; BG, Soil β-glucosidase; NAG + LAP, the sum of soil *N*-acetyl-β-d-glucosidase and soil leucine aminopeptidase; and ACP, soil acid phosphatase. Different lowercase letters indicate significant differences between P addition rates at identical N addition rates. Different capital letters indicate significant differences between N addition rates at identical P addition rates (*p* < 0.05).

**Figure 4:**
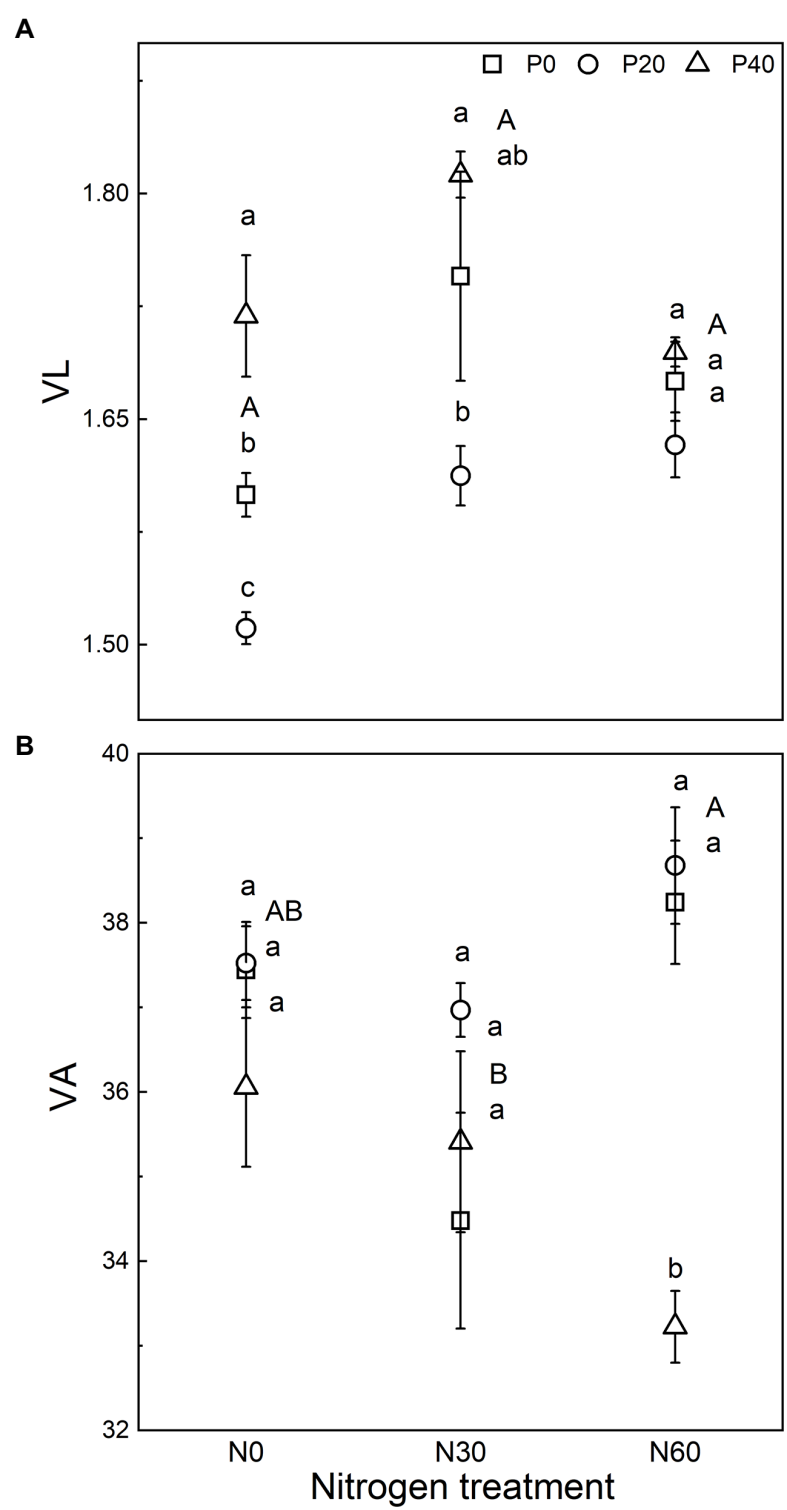
Vector analysis of enzyme activity to indicate the effect of nitrogen (N) deposition and phosphorus (P) addition on the relative nutrient limitation of the soil. **(A)** Vector length indicates soil C limitation. **(B)** Vector angle indicates the relative nutrient limitation of N or P. VL, vector length; VA, vector angle. N0, 0 kg N ha^−2^ year^−1^; N30, 30 kg N ha^−2^ year^−1^; N60, 60 kg N ha^−2^ year^−1^; P0, 0 mg P kg^−1^; P20, 20 mg P kg^−1^; and P40, 40 mg P kg^−1^. Different lowercase letters indicate significant differences between P addition rates at identical N addition rates. Different capital letters indicate significant differences between N addition rates at identical P addition rates (*p* < 0.05).

We analyzed the stoichiometry of BG, NAG + LAP, and ACP activity (Figure 3). The ln(BG):ln(NAG + LAP) ratio, an indicator of potential C:N acquisition activity, was less than 1 under the N0, P20, N30, N30 + P20, and N60 + P40 treatments. The corresponding C:P ratio, represented by the ratio of ln(BG):ln(ACP) activity under all treatments, was greater than 1. The N:P ratio, ln(NAG + LAP):ln(ACP), activity under all treatments was also greater than 1 (Figure 3). In general, compared with the control, E_C:N_ decreased under the P20 treatment (*p* < 0.05), and E_C:P_ increased under the P40 treatment (*p* < 0.05). The N60 treatment increased the E_C:N_ ratio (*p* < 0.05). Under the N30 treatment, P40 treatment significantly increased the E_C:N_ ratio (*p* < 0.05). Under the N60 treatment, P40 treatment reduced E_C:N_ and increased E_C:P_ and E_N:P_ (*p* < 0.05). The vector analysis showed that compared with the control, P20 addition significantly reduced VL values, while P40 addition significantly increased VL values (*p* < 0.05; Figure 4). The VA values were less than 45° under all treatments, indicating that the microorganisms were N-restricted.

### Effect of N and P Addition on Soil Enzyme Performance

**Figure 5:**
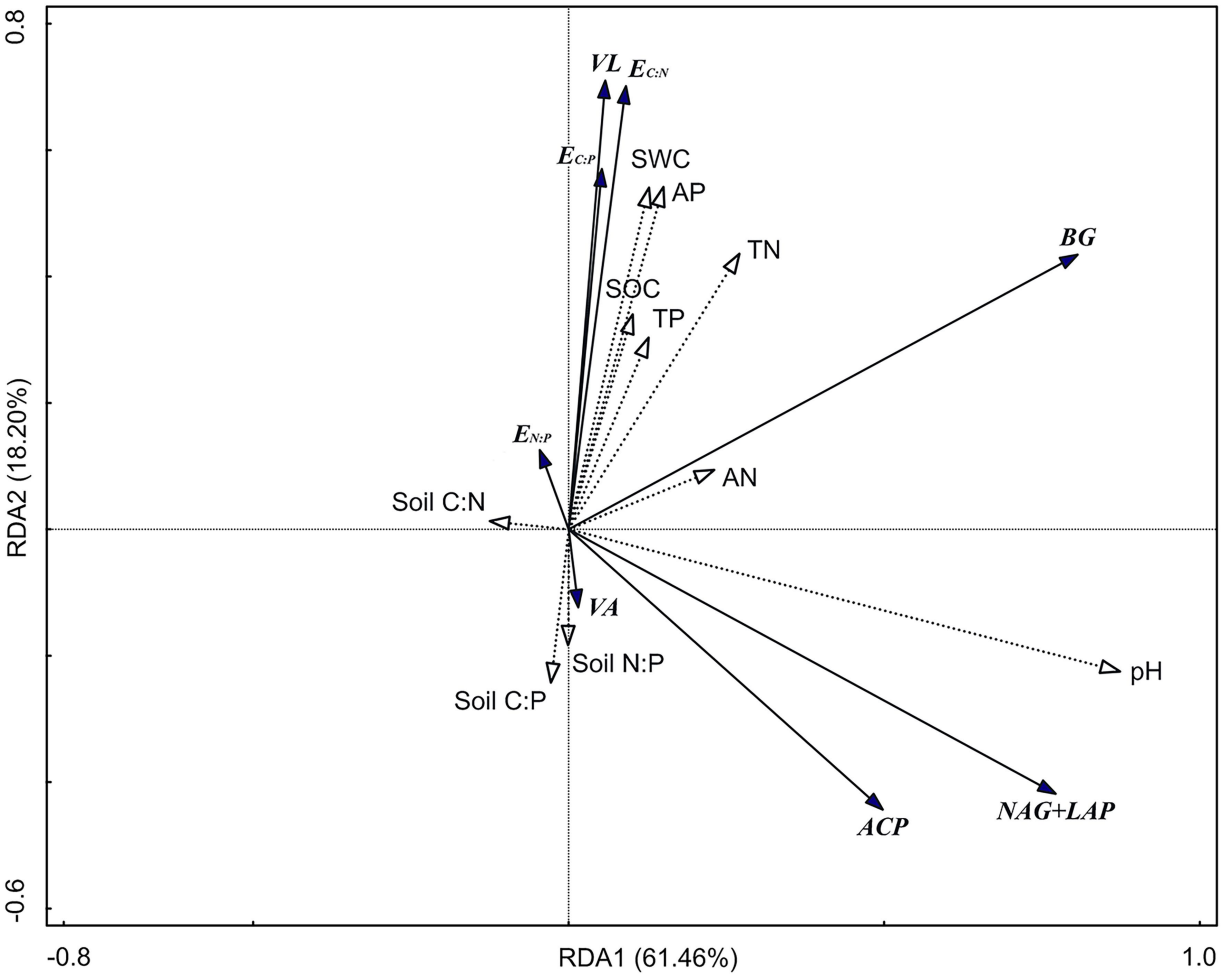
Redundancy analysis (RDA) of soil enzyme activity and enzyme stoichiometric ratio. BG, Soil β-glucosidase; NAG + LAP, the sum of soil *N*-acetyl-β-d-glucosidase and soil leucine aminopeptidase; and ACP, soil acid phosphatase. E_C:N_, ln(BG):ln(NAG + LAP); E_C:P_, ln(BG):ln(ACP); E_N:P_, ln(NAG + LAP):ln(ACP); VL, vector length; VA, vector angle; SWC, soil water content; SOC, soil organic carbon; TN, total nitrogen; AN, available nitrogen; TP, total phosphorus; AP, available phosphorus; Soil C:N, the ratio of soil SOC to TN; Soil C:P, the ratio of soil SOC to TP; and Soil N:P, the ratio of soil TN to TP.

The RDA results show (Figure 5) that the first axis explains 61.46% of the variables, and the second axis explains 18.20% of the variables. Soil pH (47.8%; *F* = 22.9, *p* = 0.002), TN (10%; *F* = 5.7, *p* = 0.004), and AP (8.5%; *F* = 5.8, *p* = 0.004) were significant factors affecting soil enzyme activity and the enzyme stoichiometric ratio (*p* < 0.01). There was a significant positive correlation between pH and BG, NAG + LAP, and ACP activities (*p* < 0.01; Table 3). Moreover, there was a significant positive correlation between TN and BG activity (*p* < 0.05). TP was significantly positively correlated with E_C:P_, E_N:P_, and VL (*p* < 0.05) and significantly negatively correlated with VA (*p* < 0.05).

**Table 3:** Pearson correlation analysis between soil enzyme activity and enzyme stoichiometric ratio and soil factors.

	BG	NAG + LAP	ACP	E_C:N_	E_C:P_	E_N:P_	VL	VA	pH	SWC	SOC	TN	AN	TP	AP
BG	1	0.435^*^	0.237	0.525^**^	0.319	−0.043	0.423^*^	0.013	0.607^**^	0.336	0.230	0.408^*^	0.227	0.232	0.355
NAG + LAP		1	0.613^**^	−0.473^*^	−0.189	0.067	−0.317	−0.096	0.768^**^	−0.123	−0.067	0.026	0.138	−0.021	−0.106
ACP			1	−0.143	−0.799^**^	−0.719^**^	−0.752^**^	0.703^**^	0.521^**^	−0.316	−0.009	−0.043	0.082	−0.333	−0.310
E_C:N_				1	0.278	−0.338	0.536^**^	0.338	−0.089	0.374	0.209	0.360	0.135	0.053	0.291
E_C:P_					1	0.808^**^	0.960^**^	−0.809^**^	−0.076	0.434^*^	−0.002	0.231	0.025	0.423^*^	0.447^*^
E_N:P_						1	0.612^**^	−0.998^**^	−0.064	0.203	−0.126	0.004	−0.055	0.392^*^	0.262
VL							1	−0.612^**^	−0.106	0.487^**^	0.064	0.302	0.052	0.400^*^	0.490^**^
VA								1	0.030	−0.211	0.125	0.001	0.056	−0.396^*^	−0.266
pH									1	−0.134	−0.089	−0.069	−0.055	0.113	0.046
SWC										1	0.495^**^	0.524^**^	0.450^*^	0.209	0.215
SOC											1	0.620^**^	0.650^**^	0.256	0.256
TN												1	0.751^**^	−0.192	−0.141
AN													1	−0.214	−0.185
TP														1	0.893^**^
AP															1

BG, Soil β-glucosidase; NAG + LAP, the sum of soil *N*-acetyl-β-d-glucosidase and soil leucine aminopeptidase; and ACP, soil acid phosphatase. E_C:N_, ln(BG):ln(NAG + LAP); E_C:P_, ln(BG):ln(ACP); E_N:P_, ln(NAG + LAP):ln(ACP); VL, vector length; VA, vector angle; SWC, soil water content; SOC, soil organic carbon; TN, total nitrogen; AN, available nitrogen; TP, total phosphorus; and AP, available phosphorus.

* *p* < 0.05;

** *p* < 0.01.

## Discussion

### Effect of Nitrogen Addition on Soil Enzyme Activity

We examined the effect of N and P addition on the soil enzymes involved in C, N, and P cycling using a 3-year simulation of N deposition and P addition in a Chinese fir forest. The results suggested that N addition had a significant effect on soil EEA in the studied Chinese fir forest. We observed that N addition increased BG activity, which is involved in C-cycling, and decreased NAG + LAP activity, which is involved in soil N cycling. Our results were in agreement with previous reports that the addition of exogenous nutrients changes the activity of extracellular enzymes (Li et al., 2021b; Shi et al., 2021; Xiao et al., 2021). These results can be explained by the resource allocation theory of enzyme production that N addition inhibits the activity of N-cycling enzymes and increases the activity of other enzymes (Sinsabaugh and Moorhead, 1994; Allison and Vitousek, 2005). However, N addition had no significant effect on soil ACP enzyme activity, which is inconsistent with other studies showing that N addition enhances soil phosphatase activity (Saiya-Cork et al., 2002; Wang et al., 2011; Fan et al., 2020).

Our results also showed that the activity of soil BG, NAG + LAP, and ACP was positively correlated with soil pH, which is consistent with previous reports (Sinsabaugh et al., 2008; Dong et al., 2015; Xiao et al., 2020), and shows that N application affected soil enzyme activity by reducing soil pH. Many studies have found significant responses of soil and microbial activity to N addition and suggest that the negative effects of N on soil microorganisms are mainly driven by soil acidification (Treseder, 2008; Liu and Greaver, 2010; Chen et al., 2016). For instance, Li et al. (2021b) reported that N fertilization increased soil NH_4_^+^, and activity of BG and ACP. Wu et al. (2019) observed that N addition changed soil pH and the content of NH_4_^+^-N and AP, which elicited a negative effect on microbial activity. Changes in soil pH and soil C:P are likely to affect the ACP activity and its response to N addition (Zhang et al., 2018; Li et al., 2021c), although ACP activity was not significantly affected by N addition in our study. The increased soil AP content with nitrogen addition suggests that N addition affects the soil AP and less P is obtained from microorganisms, which results in the no obvious change in ACP activity. Another possible explanation for the different effects of N addition on soil enzyme activity is that the trees in our plots were in the young, fast-growing stage with a large demand for N (Wu et al., 2020), and the soil had not yet reached the N-saturation state.

In addition, the soil enzyme C:N and C:P ratios were generally altered by N addition. N addition increased the ratios of soil enzymes E_C:N_ and E_C:P_ (Figure 3), and VL (Figure 4A), which implies that C will become limiting relative to N, and that microorganisms will produce more C-cycling enzymes to satisfy metabolic requirements (Chen et al., 2018b). N fertilization-induced soil acidification may also lead to reinforced microbial C-limitation (Ning et al., 2021). As microorganisms change the way they allocate resources to adapt to environmental changes (Schimel et al., 2007), N addition alters the microbial nutrient acquisition strategy and promotes the secretion of C-cycle enzymes. Taken together, N addition mitigated microbial N-limitation and enhanced microbial C-limitation.

### Effect of P Addition on Soil Enzyme Activity

Phosphorus (P) is an important limiting factor in subtropical ecosystems and plays an important role in regulating the C and N cycles (Vance et al., 2003). Given that P showed maximum limitation in most subtropical ecosystems (Hou et al., 2020; Du et al., 2021), we expected that P addition would enhance the soil enzyme activity. Indeed, soil enzyme activity related to C, N, and P cycling all responded to P addition. Furthermore, we found a positive correlation between the effect of P addition on enzyme activity and soil pH (Table 3). These results are consistent with previous studies showing that P addition increases soil microbial activity and the secretion of various enzymes by altering the characteristics of the soil. For instance, Lin et al. (2019) found that P addition enhanced soil enzymes (BG and NAG) in a natural subtropical evergreen broad-leaved forest, and Yuan et al. (2020) found that P addition increased BG enzyme activity in tropical coastal forests.

We observed the ratio of the natural logarithm of C-, N-, and P-acquiring enzymes is approximately 1.3:1.3:1 in this study, which diverged from the ratio of 1:1:1 on a global scale (Sinsabaugh et al., 2009). Cui et al. (2021) revealed the relationships of C:N:P-acquiring enzyme activities far from the widely recognized mean ratio of 1:1:1 in different ecosystems. Low-concentration P fertilizers addition resulted in the drift of the ratio to 1.2:1.3:1, indicating that microorganisms are still co-limited by C and N (Yang et al., 2020). Moreover, the RDA results also showed that the VL values were reduced and the VA values were lower than 45° after P20 addition. This indicates that the application of low amounts of P alleviated the limiting influence of microbial-available C but had no significant effect on N-limitation. These results indicate that the soil was still relatively deficient in C and N after the addition of low concentrations of P, which may be partly explained by the associated increase in microbial activity, which reduced soil C retention (Chen et al., 2021). Increased E_C:P_ ratios show that soil microbial activity related to C-cycling enzymes was inhibited after the addition of high concentrations of P. Increased VL values also show that P40 addition intensified soil C restriction. This indicates that the addition of high concentrations of P lead to insufficient soil C availability and increase the microbial demand for C. Therefore, our results indicate that the soil microorganisms changed their nutrient acquisition strategy and increased BG production to alleviate C restriction.

Furthermore, the input of a large amount of exogenous P reduces the microbial demand for P, thereby reducing the secretion of phosphatase (Olander and Vitousek, 2000; Allison and Vitousek, 2005; Marklein and Houlton, 2012; Shi et al., 2021). However, we did not find a significant effect of P40 on ACP activity or the ratio of ACP:MBC. Although the mechanisms underlying the responses of soil ACP to P addition are unclear, soil pH and C:P are likely to affect the activity of ACP and its response to P addition. The ratios of E_C:P_ increase with the addition of high concentrations of P, providing additional evidence of no significant microbial production of phosphatase to acquire P in Chinese fir forests. In addition, the sources of ACP may be soil microorganisms, mycorrhiza, and roots, but we still have limited knowledge to determine the main source (McGill and Cole, 1981). Piotrowska and Wilczewski (2012) and Stursova et al. (2006) found that the activity of various soil enzymes was largely affected by tree species after the application of various nutrients, suggesting that ACP enzymes may have been derived from plants under high P concentration treatments, which needs to be further studied.

### Interaction of N and P Additions on Soil Enzyme Activity

In this study, N + P addition significantly affected the activity of BG, NAG + LAP, and ACP, indicating that these enzymes are easily stimulated by substrates (Dong et al., 2015). We found that the interactive effect of N and P addition on soil characteristics (soil pH, SOC, TN, and AP) was significant, most likely due to the significant response of soil enzyme activity (Kuypers et al., 2018; Dai et al., 2020) to N and P addition. Our results are consistent with those of Dong et al. (2015) who reported that N and P co-addition had significant effects on soil extracellular enzymes involved in C, N, and P cycling in subtropical forests, indicating that soil microorganisms show differential demand for N and P at different concentrations of N and P addition.

The observed changes in soil properties may be a major explanatory factor for the significant responses of soil enzymes to N and P addition. Indeed, soil nutrient availability affects the utilization efficiency of nutrients by microorganisms and stimulates changes in enzyme stoichiometry (Zheng et al., 2015; Zhang et al., 2018). In our study, high N and various P treatments reduced the soil TN content and increased the TP and AP content, and the changes in the ratio of enzyme activity to MBC were consistent with the changes in enzyme activity. This indicates a negative feedback effect between soil enzyme activity and high nutrient concentrations (McGill and Cole, 1981). The vector analysis shows that the application of low amounts of P under the condition of high N alleviated N-limitation, while the application of high P increased the N restriction. The increased SOC content and the BG:MBC ratio under the N30 + P40 treatment indicates that the low N and high P concentration treatments stimulated BG enzyme production by microorganisms related to C acquisition (Allison et al., 2010). Ullah et al. (2019) found that soil enzyme activity related to C cycling is affected by soil organic matter content. In addition, the redundancy analysis provides strong evidence that the low N and high P concentration treatments aggravated soil C-limitation. This indicates that N and P addition changed the soil nutrient content, which stimulated a change in the microbial nutrient acquisition strategy, whereby soil enzyme activity was altered to adapt to nutrient limitation (Schimel et al., 2007).

Soil pH is an important factor that influences soil enzyme activity (Burns et al., 2013; Wang et al., 2014; Li et al., 2021b), with soil enzymes showing a clearly defined optimal pH range (Frankenberger and Tabatabai, 1980). Although we did not find any significant negative relationships between soil pH and the E_C:N_, E_C:P_, and E_N:P_ ratios, soil enzyme activity (BG, NAG + LAP, and ACP) and soil pH were positively correlated, which is consistent with the results of Li et al. (2021b). Specifically, the higher N and P inputs caused changes in soil pH, which affected the migration of P and ultimately led to changes in soil P content and E_C:P_ and E_N:P_ ratios. These effects of soil pH on soil enzyme activity reflect the influence of nutrient addition on soil properties and microbial communities (Chen et al., 2019).

N and P addition likely provided more exogenous resources to the soil microorganisms, causing changes in the C:N:P ratio that likely affected soil N and P availability (Tu et al., 2014) and resulting in the shift to N-limitation and C-limitation. These changes of microorganisms are linked to the soil characteristics (soil pH, TN, and AP, which explained most of the variation in soil EES), influencing the metabolism of microorganisms (Yang et al., 2020). Under projected global change scenarios, the expected EEA changes could have the important impacts on the nutrient cycling in the soil. Indeed, our study provides evidence that soil EES can be used to interpret the changes in microbial nutrient limitation resulting from inputs of exogenous resources. More broadly, the response of microbes under N deposition and P addition responsible for soil C acquisition will help predict ecosystem resilience to future global changes. Overall, our study should help develop management strategies by controlling soil C and nutrient cycling for the improvement of plantation productivity of Chinese fir forests under scenarios of increasing N deposition. In addition, seasonal variation and biological interactions should have potential impact on the soil enzyme activity. Therefore, future studies are needed to investigate the responses of soil enzyme activity to N deposition in the site-specific seasonal changes, plants, soil, and microbial characteristics.

## Conclusion

Our results show that N addition alone increased BG activity and BG:MBC, decreased NAG + LAP activity, and increased the E_C:N_ and E_C:P_ ratios, thereby alleviating microbial N-limitation and aggravating microbial C-limitation. P addition alone increased enzyme activity. N and P co-addition significantly affected EEA and EES. Soil pH was the main factor influencing enzyme activity overall, and the stoichiometric relationships of enzymes were coupled with soil pH, TN, and AP. These results indicate that changes in soil characteristics induced by N and P inputs influence the activities of soil microorganisms and result in changes in microbial resource acquisition strategies. Our results provide useful insights into the improvement of *C. lanceolata* plantation productivity by controlling soil C and nutrient cycling in forests subject to increasing N deposition.

## Data Availability Statement

The raw data supporting the conclusions of this article will be made available by the authors, without undue reservation.

## Author Contributions

XS designed the experiments and had the overall responsibility for the study. BG and QL performed the experiments and analyzed the data. ML, BG, WX, and XS wrote the draft. All authors contributed to the article and approved the submitted version.

## Funding

The study was funded by the National Natural Science Foundation of China (NSFC; grant no. 31971623), the National Key Research and Development Program of China (grant no. 2016YFD0600201), and the Ten Thousand People Program of Zhejiang Province (grant no. 2018R52027).

## Conflict of Interest

The authors declare that the research was conducted in the absence of any commercial or financial relationships that could be construed as a potential conflict of interest.

## Publisher’s Note

All claims expressed in this article are solely those of the authors and do not necessarily represent those of their affiliated organizations, or those of the publisher, the editors and the reviewers. Any product that may be evaluated in this article, or claim that may be made by its manufacturer, is not guaranteed or endorsed by the publisher.
